# N-terminal pro-B-type natriuretic peptide and microsize myocardial infarction risk in the reasons for geographic and racial differences in stroke study

**DOI:** 10.1186/s12872-018-0806-4

**Published:** 2018-04-16

**Authors:** Madeline R. Sterling, Raegan W. Durant, Joanna Bryan, Emily B. Levitan, Todd M. Brown, Yulia Khodneva, Stephen P. Glasser, Joshua S. Richman, George Howard, Mary Cushman, Monika M. Safford

**Affiliations:** 1 Division of General Internal Medicine, Department of Medicine, Weill Cornell Medical College, 1300 York Avenue, P.O. Box 46, New York, N.Y 10065 USA; 2 Division of Preventive Medicine, Department of Medicine, University of Alabama at Birmingham School of Medicine, Birmingham, AL USA; 3 Department of Epidemiology, University of Alabama at Birmingham School of Public Health, Birmingham, AL USA; 4 Division of Cardiovascular Disease, Department of Medicine, University of Alabama at Birmingham School of Medicine, Birmingham, AL USA; 5 Department of Internal Medicine, University of Kentucky College of Medicine, Louisville, KY USA; 6 Department of Surgery, University of Alabama at Birmingham School of Medicine, Birmingham, AL USA; 7 Department of Biostatistics, University of Alabama at Birmingham School of Public Health, Birmingham, AL USA; 8 Departments of Medicine and Pathology, Larner College of Medicine at the University of Vermont, Burlington, VT USA

**Keywords:** Coronary heart disease, B-type natriuretic peptide, Mircosize myocardial infarction, Racial disparities, Cohort study

## Abstract

**Background:**

N-terminal pro B-type peptide (NT-proBNP) has been associated with risk of myocardial infarction (MI), but less is known about the relationship between NT-proBNP and very small non ST-elevation MI, also known as microsize MI. These events are now routinely detectable with modern troponin assays and are emerging as a large proportion of all MI. Here, we sought to compare the association of NT-proBNP with risk of incident typical MI and microsize MI in the REasons for Geographic and Racial Differences in Stroke (REGARDS) Study.

**Methods:**

The REGARDS Study is a national cohort of 30,239 US community-dwelling black and white adults aged ≥ 45 years recruited from 2003 to 2007. Expert-adjudicated outcomes included incident typical MI (definite/probable MI with peak troponin ≥ 0.5 μg/L), incident microsize MI (definite/probable MI with peak troponin < 0.5 μg/L), and incident fatal CHD. Using a case-cohort design, we estimated the hazard ratio of the outcomes as a function of baseline NT-proBNP. Competing risk analyses tested whether the associations of NT-proBNP differed between the risk of incident microsize MI and incident typical MI as well as if the association of NT-proBNP differed between incident non-fatal microsize MI and incident non-fatal typical MI, while accounting for incident fatal coronary heart disease (CHD) as well as heart failure (HF).

**Results:**

Over a median of 5 years of follow-up, there were 315 typical MI, 139 microsize MI, and 195 incident fatal CHD. NT-proBNP was independently and strongly associated with all CHD endpoints, with significantly greater risk observed for incident microsize MI, even after removing individuals with suspected HF prior to or coincident with their incident CHD event.

**Conclusion:**

NT-proBNP is associated with all MIs, but is a more powerful risk factor for microsize than typical MI.

**Electronic supplementary material:**

The online version of this article (10.1186/s12872-018-0806-4) contains supplementary material, which is available to authorized users.

## Background

B-type natriuretic peptide (BNP) and its N-terminal fragment (NT-proBNP) are cardiac-derived secretory hormones with diuretic, vasodilatory, and natriuretic properties [1]. Although it is commonly used in assessing patients with heart failure (HF), NT-proBNP is independently associated with risk of coronary heart disease (CHD) as well as adverse outcomes among patients with ST-elevation myocardial infarction (STEMI). [2–5] Recently, the prognostic implications of NT-proBNP have been extended to patients with non–ST-elevation MI (NSTEMI) and those with stable CHD presenting with chest pain [6, 7].

While NT-proBNP is associated with MI in general, less is known about the relationship between NT-proBNP and the emerging disorder of very small NSTEMI, or microsize MI. Microsize MI has modest peak troponin elevations that in the past were considered to have uncertain clinical relevance [8]. Because of the wide implementation of sensitive troponin assays, microsize MIs are now increasingly recognized and have been shown to be associated with elevated long term CHD risk [8, 9]. A better understanding of the similarities and differences in risk factors between microsize and typical MIs is needed to guide risk stratification and optimize clinical management [10–13].

Prior to the widespread use of highly sensitive troponin assays, studies demonstrated an association of NT-proBNP with the incidence of typical MIs, but were limited in the ability to differentiate typical from microsize MIs. Therefore, we hypothesized that there would be an association between baseline levels of NT-proBNP and risk of incident microsize MIs. We conducted this study in the national Reasons for Geographic and Racial Differences in Stroke (REGARDS) cohort study.

## Methods

### REGARDS cohort study procedures

Details of the REGARDS study have been described previously [14]. Briefly, REGARDS is a prospective cohort study evaluating racial and geographic disparities in stroke and MI. 30,239 English-speaking, community dwelling U.S. adults ≥ 45 years of age were recruited between January 2003 and October 2007 by mail and telephone. Blacks and residents of the stroke belt were oversampled [14]. Participants completed a telephone interview ascertaining medical history followed by an in-home examination assessing blood pressure levels, height and weight, ECGs, anthropomorphic measures, and blood and urine samples along with a medication inventory. Blood and urine samples were processed at the REGARDS Central Laboratory at the University of Vermont. Phlebotomy was performed by trained personnel after a 10–12 h fast, using standardized procedures. Within 2 h of collection, samples were centrifuged and shipped on ice to the University of Vermont, where, upon arrival, samples were centrifuged at 30,000 × G and 4 °C, and either analyzed (general chemistries) or stored at − 80 °C [15, 16].

At six month intervals, participants are contacted by phone to ask about hospitalizations and general health status, with medical record retrieval for suspected coronary events. The study protocol was reviewed and approved by the University of Alabama at Birmingham Institutional review board and all participants provided written informed consent.

### Primary outcome(s)

For CHD events, medical records were adjudicated by expert clinicians based on published guidelines [17, 18] considering clinical signs and symptoms consistent with ischemia; a rising and/or falling pattern of troponin over ≥ 6 h with a peak at least twice the upper limit of normal; and/or ECG or other imaging findings consistent with ischemia based on the Minnesota code [19]. Cases where the clinical presentation was equivocal or where the troponin rise was less than twice the upper limit of normal were adjudicated to be possible MIs and were not included as outcomes in this analysis. Cases with low-level troponin elevations without a rising and/or falling pattern over ≥ 6 h or with a non-ischemic cause of troponin elevation were not considered to be MIs. Elective and urgent coronary revascularization procedures were not included in the definition of MI and we classified MIs caused by an invasive procedure as procedure related. Only definite or probable MIs were considered as MIs. Definite MIs included cases with diagnostic enzymes or electrocardiogram and probable MIs included cases with elevated but not diagnostic (ie, equivocal) enzymes with a positive but not diagnostic electrocardiogram or, if enzymes were missing, a positive electrocardiogram in the presence of ischemic signs or symptoms.

As other REGARDS studies have done, 0.5 μg/L was used as the threshold of peak troponin to define microsize MI [20, 18]. This definition of microsize MI (peak troponin < 0.5 μg/L) has been previously used by Safford et al. (2013) and is based on clinical practice as well as an analyses of troponin assay 99th percentiles and 10% CV levels available on the International Federation of Clinical Chemists website [21]. All other adjudicated definite or probable MIs were considered to be typical MIs (peak troponin ≥ 0.5 μg/L) [20, 21].

Since death at the first presentation with CHD remains common, an additional endpoint included total incident fatal CHD (deaths caused by CHD including sudden deaths and death within 28 days of an adjudicated MI). Interviews with next of kin, the National Death Index, [14] death certificates, medical records, and autopsy reports were used to adjudicate cause of death.

### NT-proBNP

Baseline NT-Pro BNP was measured using the Roche Elecsys analyzer (Roche Diagnostics Indianapolis, IN) which utilizes an electrochemiluminescence immunoassay (inter-assay coefficient of variation < 5%) [22].

### Covariates

Demographic data included self-reported age, sex, race (black or white), education (less than high school and high school or higher), annual income (less than $20,000 and $20,000 and above). Region of residence was classified as being a resident of the stroke buckle (the coastal regions of North and South Carolina and Georgia), the stroke belt (the remainder of North and South Carolina and Georgia, plus Alabama, Mississippi, Louisiana, Arkansas, and Tennessee), or not a stroke belt resident (the remaining 40 contiguous United States). Clinical CHD risk factors included diabetes, defined as fasting blood glucose ≥126 mL/dL (or glucose > 200 mL/dL for those failing to fast) or oral hypoglycemic or insulin use; systolic blood pressures based on the average of 2 standardized blood pressure measurements (continuous variables in mm Hg); body mass index (BMI) based on measured height and weight; cigarette smoking (current smoking versus never or former smokers); albumin-to-creatinine ratio (ACR), which was log transformed for the analysis; high sensitivity c-reactive protein (hsCRP), which was also log transformed; and high-density lipoprotein (HDL) and total cholesterol. Estimated glomerular filtration rate (eGFR) was calculated using the Chronic Kidney Disease (CKD) Epidemiology Collaboration formula, [23] with CKD defined as eGFR as < 60 ml/min/1.73 [2]. Medication use included aspirin (yes or no), statins (yes or no), and antihypertensive medications (yes or no).

### Case-cohort design

The case-cohort study design minimizes the cost of expensive biomarker assays without compromising the power advantage of large cohort studies [24, 25]. Here, the study was comprised of all incident acute CHD cases through 12/31/2010 and a stratified random sample of participants free of CHD at baseline and not on dialysis (the sub-cohort). The sub-cohort random sample was selected as previously described using stratified random sampling in 20 age (45–54, 55–64, 65–74, 75–84, ≥ 85 years), race (black and white), and sex strata [26].

### Statistical analysis

Participant characteristics were compared across tertiles of NT-proBNP within the cohort random sample using appropriate sampling weights. Analysis of variance and Χ^2^ tests were used to evaluate significant differences.

Using Cox regression for case-cohort studies, [25] with Barlow and Prentice sampling weights, [27] we performed competing risk analyses as described by Lunn and McNeil [28] incorporating the sub-cohort sampling weights to test whether the association between NT-proBNP and the risk of incident microsize MI differed from 1) that of typical MI and 2) while also accounting for incident fatal CHD death. Hazard ratios (HR) and 95% confidence intervals (CI) were calculated for the outcomes defined above as a function of NT-proBNP in crude models and models adjusted for demographics (age, race, sex, income, education, geographic region of residence), Framingham risk factors (smoking, systolic blood pressure, diabetes, HDL, total cholesterol), and additional CVD risk factors (BMI, hsCRP, ACR, eGFR, medications). NT-proBNP was analyzed in tertiles with the lowest tertile serving as the reference group. In the analysis accounting for incident fatal CHD death, MI events followed by death within 28 days were classified as CHD death, effectively comparing the association between NT-proBNP and three separate outcomes: nonfatal typical MI, nonfatal microsize MI, and fatal CHD.

Because elevated BNP may be associated with HF, we repeated all analyses excluding individuals with NT-proBNP levels above the age-specific normal range (> 125 pg/mL if < 75 years old and > 450 pg/mL if ≥75 years old), with an adjudicated HF hospitalization prior to their MI event or CHD death, or who had HF during the incident MI hospitalization.

In all analyses, a 2-tailed *P* value < 0.05 was considered statistically significant. Additionally, 10% of individuals had at least one missing value for covariates, thus final analyses were conducted with multiple imputation using chained equations [29]. Covariates with the largest amount of missing values were ACR (4%) and hsCRP (2%). Analyses were conducted using SAS software version 9.4 (SAS Institute, Cary, NC) and Stata 14 (Stata Corp, College Station, TX).

## Results

### Baseline characteristics of the study participants

Of the 30,239 REGARDS participants, we excluded 569 who were missing follow up, leaving 29,670 from which 1104 were drawn for the random sub-cohort. Of these, 50 were excluded for missing NT-proBNP, 23 for laboratory inconsistencies, 182 for a history of CHD at baseline, and 2 on dialysis. A final analytic sub-cohort of 847 was used for this analysis (Additional file 1: Fig. S1). Over a median of 5.0 (IQR: 3.5, 6.3) years of follow-up time, there were 454 incident MI cases, of which 315 were typical MIs and 139 were microsize MIs. The median time to event for microsize MI was 2.7 (IQR: 1.3, 5.5) and the median time to event for typical MI was 2.2 (1.1, 3.7). There were also 195 incident fatal CHD cases. Twenty-one of the participants who experienced incident MI or fatal CHD were in the randomly selected sub-cohort.

Baseline characteristics of sub-cohort random sample participants by tertile of NT-proBNP are shown in Table 1. Higher NT-proBNP was characterized by female sex, white race, older age, lower educational attainment, lower annual income, higher risk factor levels, and a greater burden of medical illness.

**Table 1:** Baseline Characteristics of Study Participants in the Cohort Random Sample by Tertiles of NT-proBNP

	Brain Natriuretic Peptide (N-terminal pro BNP), tertiles of pg/mL			
Characteristics*	< 42.5	42.5–115.5	≥115.5	*p*-value †
Total participants, n	278 ^‡^	280^‡^	289^‡^	
Male, n (%)	168 (60.4)	119 (42.5)	101 (35.0)	< 0.001
African American, n (%)	168 (56.8)	129 (46.1)	142 (49.1)	0.03
Age in years, mean (SD)	59.7 ± 9.5	65.9 ± 11.2	74.5 ± 10.9	< 0.001
Less than High School education, n (%)	28 (10.1)	38 (13.6)	61 (21.1)	0.001
Annual Income, n (%)				0.001
< $20,000	38 (13.7)	46 (16.4)	71(24.6)	
≥$20,000	208 (74.8)	193 (68.9)	157 (54.3)	
Declined to report	32 (11.5)	41 (14.6)	61 (21.1)	
Current smoking, n (%)	46 (16.6)	41 (14.7)	33 (11.5)	0.21
Systolic blood pressure, mmHg, mean (SD)	125 ± 14.8	127 ± 17.4	130.8 ± 18.0	< 0.001
High density lipoprotein, mg/L, mean (SD)	50.0 ± 14.7	53.1 ± 17.0	54.0 ± 18.7	0.005
Total cholesterol, mg/L, mean (SD)	196 ± 41	192 ± 37	186 ± 37	< 0.001
Diabetes, n (%)	52 (19.1)	49 (17.8)	59 (20.6)	0.67
Body mass index, kg/m^2^, mean (SD)	29.8 ± 5.7	29.1 ± 6.5	27.6 ± 5.5	< 0.001
C-reactive protein mg/L, median (IQR)	1.9 [0.9–5.0]	2.1 [0.9–4.7]	2.2 [1.1–4.7]	0.37
Urinary albumin-to-creatinine ratio > 30 mg/g, n (%)	14 (5.1)	30 (11.2)	65 (23.6)	< 0.001
Estimated glomerular filtration mL/min/1.73 m^2^ rate < 60^§^, n (%)	10 (3.6)	25 (9.0)	70 (24.5)	< 0.001
Use of hypertensive medication, n (%)	112 (40.4)	128 (45.9)	173 (60.5)	< 0.001
Use of statins, n (%)	73 (26.5)	69 (24.9)	78 (27.2)	0.82
Regular use of aspirin, n (%)	97 (35.0)	95 (34.0)	117 (40.6)	0.21

* Characteristics within the cohort random sample; missing values included use of statins (*n* = 7), use of hypertensive medication (*n* = 5), estimated glomerular filtration rate (eGFR) (*n* = 7), baseline diabetes (*n* = 11), C-reactive protein (*n* = 23), aspirin use (*n* = 2), urinary albumin-to-creatinine ratio (*n* = 32), systolic blood pressure (*n* = 5), high density lipoprotein (*n* = 13), cholesterol (n = 7), and body mass index (n = 7). † *P*-values from analysis of variance for continuous variables and Pearson chi square for categorical variables. ‡ Data weighted to the full cohort for analysis. Tertiles based off of weighted sample. § eGFR < 60 mL/min/1.73 m^2^ = chronic kidney disease (CKD)

### Associations of NT-proBNP with microsize and typical MIs

The cause-specific hazard ratios (CHR) for microsize and typical MIs by tertile of NT-proBNP are shown in Table 2. A significant difference in the association between NT-proBNP and the risk of microsize versus typical MI was seen in unadjusted and adjusted models (p for equality = 0.03). In the unadjusted model, participants in the highest tertile of baseline NT-proBNP compared to the lowest had higher risk of incident microsize MI (CHR 7.06, CI: 3.96–12.57) compared to incident typical MI (CHR 2.86, CI: 2.01–4.05). These results were similar with full adjustment (NT-proBNP and microsize MI adjusted CHR [aCHR] 6.83 (CI: 3.52–13.25) and typical MI aCHR 2.63 (CI: 1.63–4.33) with a p for equality of 0.03 (Table 2).

**Table 2:** Cause- Specific Hazard Ratios for Microsize MI and Typical MI according to Baseline Brain Natriuretic Peptide (NT-proBNP) Tertiles

	Events	Microsize MI	Events	Typical MI	*P* for equality ^a^
Model 1					
< 42.50(Ref)	16	1	73	1	**0.03**
42.5–115.5	46	**3.11 (1.70–5.69)**	97	1.41 (0.98–2.04)	
≥ 115.50	77	**7.06 (3.96–12.57)**	145	**2.86 (2.02–4.05)**	
Model 2					
< 42.50(Ref)	16	1	73	1	0.03
42.5–115.5	46	**3.18 (1.62–6.26)**	97	1.26 (0.80–1.99)	
≥ 115.5	77	**6.83 (3.52–13.25)**	145	**2.66 (1.63–4.33)**	

Model 1 = BNP alone

Model 2 = Model 1 + Demographics (age, race, sex, household income, education, geographic region of residence), additional Framingham risk factors (current smoking status, systolic blood pressure, diabetes, HDL and total cholesterol) and other CVD risk factors and covariates (body mass index, log-transformed hsCRP, log-transformed ACR and medication use

All models tested for interactions of NT-proBNP with age, race and gender

Bold *p* < 0.05

a Cause specific model using Lunn and McNeil approach for competing risk analyses

In the sensitivity analysis of the competing risk between microsize and typical MI among participants without possible HF, a total of 71 participants with microsize MIs and 133 participants with typical MIs were excluded. A significant difference in the risk of incident microsize versus typical MI by NT-proBNP tertiles persisted (p for equality = 0.05), with the association between NT-proBNP and microsize MI remaining larger across unadjusted and adjusted models, albeit of borderline statistical significance (p for equality = 0.06) (Table 3).

**Table 3:** Cause- Specific Hazard Ratios for Microsize MI and Typical MI according to Baseline Brain Natriuretic Peptide (NT-proBNP) Tertiles, Excluding Individuals with Possible Heart Failure (HF)

	Events	Microsize MI	Events	Typical MI	*P* for equality^a^
Model 1					
< 42.5(Ref)	10	1	55	1	**0.05**
42.5–115.5	25	**3.24 (1.68–6.30)**	66	1.27 (0.87–1.87)	
≥115.5	33	**5.70 (2.65–12.29)**	61	**2.46 (1.53–3.94)**	
Model 2					
< 42.50(Ref)	10	1	55	1	0.06
42.5–115.5	25	**3.17 (1.46–6.85)**	66	1.16 (0.70–1.92)	
≥115.5	33	**5.41 (2.14–13.69)**	61	**2.15 (1.07–4.32)**	

Possible heart failure (HF) defined as:

1. Baseline BNP > 125 if a participant is < than 75 years of age, or BNP > 450 if a participant ≥ 75 years

a. *N* = 362 among first sub-cohort (random sample, plus participants with CHD events)

b. *N* = 297 among second sub-cohort (random sample, plus participants with MI events)

2. Participants who had an incident MI event with concurrent prevalent HF (*n* = 18) and participants with MI and incident HF at the same date (*n* = 32)

3. Participants who had a first HF admission before an incident MI (n = 2)

Participants without MI event, but with incident HF before 01/01/2011 were censored at the time of first HF admission

Model 1 = BNP alone

Model 2 = Model 1 and Demographics (age, race, sex, household income, education, geographic region of residence), additional Framingham risk factors (current smoking status, systolic blood pressure, diabetes, HDL and total cholesterol) and other CVD risk factors and covariates (body mass index, log-transformed hsCRP, log-transformed ACR and medication use

Bold *p* < .05

^a^Cause specific model using Lunn and McNeil approach for competing risk analyses

When accounting for acute fatal CHD as a competing risk, a significant difference in the association between NT-proBNP and risk of incident non-fatal microsize MI versus non-fatal typical MI was detected in unadjusted (p for equality < 0.01) and adjusted models (p for equality = 0.03) (Additional file 1: Table S1). In the unadjusted analysis, participants in the highest tertile of baseline NT-proBNP compared to the lowest tertile had higher risk of incident non-fatal microsize MI (CHR 6.69, CI: 3.69–12.12) than typical MI (CHR 2.57, CI: 1.80–3.67) and incident fatal CHD (CHR 5.15, CI: 3.31–8.01). These results were similar with full adjustment (NT-proBNP and incident non-fatal microsize MI aCHR 6.07, CI: 3.12–11.77; typical MI aCHR 2.25, CI: 1.41–3.60; and incident fatal CHD (aCHR 4.76, CI: 2.79–8.10).

In the sensitivity analysis accounting for fatal CHD among those without possible HF, there were substantially fewer events (Additional file 1: Table S2). Similar to the main results, the unadjusted HR for non-fatal microsize MI (HR 4.69 (95% CI 2.06–10.70) was higher than for typical MI (HR 2.20 [95% CI 1.35–3.59]), but this difference was not statistically significant. These patterns were maintained after full adjustment, and were also not statistically significant.

## Discussion

In this prospective study of black and white US adults, similar to past studies, NT-proBNP was strongly associated with MI, but we observed an even stronger association between NT-proBNP and microsize MI than typical MI. The greater association between NT-proBNP and risk of microsize MI compared with typical MI persisted across multivariable models, in analyses excluding patients with potential HF, and analyses incorporating incident fatal CHD. To our knowledge, this is the first examination of the relationship of NT-proBNP with microsize MI, an entity only recently recognized as a large component of MIs in the modern era.

Increasingly sensitive troponin assays now routinely detect very small, or microsize, MIs, [30–33] which comprised 31.3% of non-fatal MI events in REGARDS and have been found to be associated with long-term CHD risk, apart from typical MIs [8]. Yet, due to variation across US laboratories, these very small events may often be missed or misclassified [21]. Work by Mills et al. (2011) suggests that misclassification may have serious clinical consequences: among patients presenting with suspected acute coronary syndrome (ACS), lowering the diagnostic threshold for MI from the previous generation troponin assay cutoff (0.20 ng/mL) to 0.05 ng/mL was associated with increased detection of MIs by 29%, increased evidence-based treatment in the group with peak troponin 0.05–0.20 ng/mL, and 50% reduction in death or recurrent MI among those with these small events [12]. The percent rise in events in the Mills study (29%) is remarkably similar to the proportion of microsize MIs in the REGARDS study (31%). These two studies, and others, [34, 35] demonstrate that failing to recognize and potentially treat microsize MI events could have profound implications for patient morbidity and mortality.

To date, the clinical characteristics of individuals who present with microsize MIs are not well described. We found that baseline levels of NT-proBNP were more strongly associated with risk of incident microsize MIs compared to typical MIs, which suggests that microsize MIs and typical MIs may differ with respect to their risk factor profiles and possibly their underlying pathophysiology. It is well known that myocardial stretch stimulates the release of NT-proBNP; NT-proBNP levels are also closely associated with vascular remodeling, inflammation, and hypertrophy in the cardiovascular system [33, 36]. Local hypoxemia and ischemia, independent of changes in left ventricular function, have been shown to induce NT-proBNP synthesis [37–39] and following tissue injury, inflammatory cytokines [40, 41] and cardiac myocytes induce BNP secretion as fibrosis [41] and vascular remodeling occurs [42]. Given the extent to which we found NT-proBNP to be associated with microsize MIs, it is plausible that these small events occur in the context of microvascular derangements of circulating factors in a way that differs from the medium vessel atherosclerotic plaque rupture that characterizes typical MIs. Recent studies further support this hypothesis by demonstrating that NT-proBNP levels are associated with small vessel disease [43, 44]. Mutlu et al. (2016) reported that in participants without cardiovascular disease, higher levels of NT-proBNP were associated with microvascular damage in the retina and retinal arterioles [45]. Additionally, Bower et al. (2015) found that higher levels of NT-proBNP were associated with incident hypertension, [46] a process that involves arteriolar narrowing, increased cardiac output, hypoxemia at the level of myocytes, and ultimately release of NT-proBNP. Finally, in a prospective study, Cushman et al. (2016), found that higher NT-pro-BNP levels were associated with incident cognitive impairment, independent of CVD and Alzheimer’s disease risk factors [16]. Together, these studies suggest that high levels of NT-proBNP may occur alongside, or in response to, systemic microvascular dysfunction in addition to, or apart from, its known association with large vessel disease.

Our findings also have important implications for the utility of NT-proBNP as a biomarker of CHD. A recent individual-participant-data meta-analysis found that among those without known baseline CVD, NT-proBNP concentration predicted first-onset HF and improved the prediction of CHD and stroke. [47] Additionally, the Dallas Heart Study demonstrated an independent association between coronary atherosclerosis burden and circulating levels of NT-proBNP [48] in a large, low-risk population without symptomatic HF or ischemia. NT-proBNP levels are independently predictive of atherosclerotic events [49, 50]; Goyal et al. (2014) found that BNP levels are related to the severity of coronary atherosclerosis such that patients with multi-vessel disease had higher BNP levels than those with one or two vessel involvement. Notably, this finding was independent of a diagnosis of unstable angina or NSTEMI. Collectively, these findings, in addition to our finding that NT-proBNP was strongly associated with both non-fatal microsize MI and fatal CHD, suggest that NT-proBNP might be used for risk stratification. However, it is important to note that major clinical trials in CHD have focused almost exclusively on typical MIs. As such, additional work is needed to identify the optimal clinical management strategies for patients surviving a microsize MI event.

### Strengths and limitations

This study’s strengths include the large, geographically and racially diverse cohort in which participants were followed prospectively. Baseline data were collected in standardized fashion and prospective data included CHD endpoints adjudicated by expert clinicians. This study is unique in that we were able to study microsize MI events, which have not been specifically reported in other cohort studies and may be difficult to capture in claims data. Finally, competing risk analyses permitted examination of the association between NT-proBNP and incident microsize MIs versus typical MIs while accounting for acute fatal CHD.

Some limitations should be considered when interpreting these findings. Only a single baseline measure of NT-proBNP was available, with varying elapsed time between baseline and the events of interest. Only black and white adults were included, potentially limiting generalizability to other races or ethnicities. We were unable to measure left ventricular ejection fraction (LVEF) among cases or controls in this study; since ischemic and dilated cardiomyopathies are often associated with elevated troponin and NT-proBNP levels, future studies on the association between NT-proBNP and microsize MIs ought to include this as a covariate in multivariable models. Our sensitivity analysis excluding those with possible HF to some extent overcomes this limitation. Finally, several variables were self-reported with known vulnerability to bias.

## Conclusion

This is the first study to examine the association of NT-proBNP with risk of microsize MI. Although baseline NT-proBNP levels were associated with microsize MIs, typical MIs, and fatal CHD, the association between NT-proBNP and risk of incident microsize MI was markedly higher than typical MI. This association persisted with adjustment for demographic and clinical covariates, excluding participants with possible HF, and accounting for fatal CHD. These results highlight the utility of NT-proBNP as a marker of CHD events, particularly the entity of microsize MIs. Our findings are concordant with past studies that suggest that these very small events represent a large proportion of MI events, but our findings build on these observations to suggest that they may differ from typical MIs, particularly with respect to risk factors and pathophysiology. Further study is needed to understand the relative contribution of traditional and non-traditional risk factors to the incidence of microsize MIs compared to typical MIs, as well as to understand differences in outcomes. If there are sufficient differences, it is possible that separate risk stratification tools may be needed, and NT-proBNP may be a candidate for inclusion in such tools. Our findings underscore that a better understanding is needed of this very large proportion of MIs in the modern era.

## Additional file

Additional file 1: **Figure S1.** Selection of the Sub-Cohort in the Reasons for Geographic and Racial Differences in Stroke (REGARDS), **Table S1.** Cause- Specific Hazard Ratios for Nonfatal Microsize MI, Nonfatal Typical MI, and Fatal CHD according to Baseline Brain Natriuretic Peptide (NT-proBNP) Tertiles, **Table S2.** Cause- Specific Hazard Ratios for Nonfatal Microsize MI, Nonfatal Typical MI, and Fatal CHD according to Baseline Brain Natriuretic Peptide (NT-proBNP) Tertiles, Excluding Individuals with Possible Heart Failure (HF). (DOCX 98 kb)
