# Supplementary material for: N-terminal pro-B-type natriuretic peptide and microsize myocardial infarction risk in the reasons for geographic and racial differences in stroke study
Source: BMC Cardiovasc Disord. 2018 Apr 16;18:66. doi: 10.1186/s12872-018-0806-4 (PMC5902876; doi:10.1186/s12872-018-0806-4)
Supplement: Supplementary file 1 — Figure S1. Selection of the Sub-Cohort in the Reasons for Geographic and Racial Differences in Stroke (REGARDS), Table S1. Cause- Specific Hazard Ratios for Nonfatal Microsize MI, Nonfatal Typical MI, and Fatal CHD according to Baseline Brain Natriuretic Peptide (NT-proBNP) Tertiles, Table S2. Cause- Specific Hazard Ratios for Nonfatal Microsize MI, Nonfatal Typical MI, and Fatal CHD according to Baseline Brain Natriuretic Peptide (NT-proBNP) Tertiles, Excluding Individuals with Possible Heart Failure (HF). (DOCX 98 kb) [file 12872_2018_806_MOESM1_ESM.docx]

**ONLINE Additional files**

Figure S1

Figure Legend: Cohort flow diagram. BNP indicates Brian natriuretic peptide; CHD, coronary heart disease; REGARDS, REasons for Geographic And Racial Differences in Stroke.

**Figure S1:** Selection of the Sub-Cohort in the Reasons for Geographic and Racial Differences in Stroke (REGARDS)

**Figure Legend:** Cohort flow diagram. BNP indicates Brian natriuretic peptide; CHD, coronary heart disease; REGARDS, REasons for Geographic And Racial Differences in Stroke.

*17 of the 454 incident MI cases are in sub- cohort. Of 17 incident MI cases, 13 are typical MIs and 4 are microsize MIs

**Table S1: Cause- Specific Hazard Ratios for Nonfatal Microsize MI, Nonfatal Typical MI, and Fatal CHD according to Baseline Brain Natriuretic Peptide (NT-proBNP) Tertiles**

| **NT-proBNP at Baseline** | **Events** | **Non-fatal Microsize MI** | **Events** | **Non-fatal Typical MI** | **Events** | **P for equality^a^**  **(Microsize and Typical MI)** | **Fatal CHD** | **P for equality^a^ (all outcomes)** |
| --- | --- | --- | --- | --- | --- | --- | --- | --- |
| **Model 1** | | | | | | | | |
| <42.50(Ref) | 15 | 1 | 71 | 1 | 33 | **0.007** | 1 | **0.018** |
| 42.50-115.50 | 38 | **2.72 (1.45- 5.11)** | 90 | **1.35 (0.93- 1.95)** | 47 |  | 1.53 (0.94- 2.51) |  |
| >115.50 | 69 | **6.69 (3.69- 12.12)** | 127 | **2.57 (1.80- 3.67)** | 115 |  | **5.15 (3.31- 8.01)** |  |
| **Model 2** | | | | | | | | |
| <42.50(Ref) | 15 | 1 | 71 | 1 | 33 | **0.029** | 1 | **0.038** |
| 42.50-115.50 | 38 | **2.67 (1.34- 5.39)** | 90 | 1.16 (0.73- 1.84) | 47 |  | 1.62 (0.93- 2.82) |  |
| >115.50 | 69 | **6.07 (3.12- 11.77)** | 127 | **2.25 (1.41- 3.60)** | 115 |  | **4.76 (2.79- 8.10)** |  |

Model 1 = BNP alone

Model 2 = Model 1 + Demographics (age, race, sex, household income, education, geographic region of residence), additional Framingham risk factors (current smoking status, systolic blood pressure, diabetes, HDL and total cholesterol) and other CVD risk factors and covariates (body mass index, log-transformed hsCRP, log-transformed ACR and medication use

**Bold p <.05**

a. Cause specific model using Lunn and McNeil approach for competing risk analyses.

**Table S2: Cause- Specific Hazard Ratios for Nonfatal Microsize MI, Nonfatal Typical MI, and Fatal CHD according to Baseline Brain Natriuretic Peptide (NT-proBNP) Tertiles, Excluding Individuals with Possible Heart Failure (HF)**

| **NT-proBNP at Baseline** | Events | Non-fatal Microsize MI | Events | Non-fatal Typical MI | Events | P for equality^a^ | Fatal CHD | P for equality^a^ |
| --- | --- | --- | --- | --- | --- | --- | --- | --- |
| **Model 1** | | | | | | | | |
| <42.5 (Ref) | 9 | 1 | 53 | 1 | 27 | 0.1820 | 1 | 0.1773 |
| 42.5-115.5 | 23 | **2.85 (1.42- 5.70)** | 64 | 1.26 (0.86- 1.86) | 30 |  | 1.41 (0.85- 2.32) |  |
| ≥115.5 | 24 | **4.69 (2.06- 10.70)** | 55 | **2.20 (1.35- 3.59)** | 47 |  | **3.85 (2.19- 6.75)** |  |
| **Model 2** | | | | | | | | |
| <42.50(Ref) | 9 | 1 | 53 | 1 | 27 | 0.1858 | 1 | 0.1666 |
| 42.5-115.5 | 29 | **2.56 (1.16- 5.67)** | 64 | 1.13 (0.69- 1.87) | 30 |  | 1.61 (0.89- 2.91) |  |
| ≥115.5 | 24 | **3.97 (1.51- 10.48)** | 55 | 1.81 (0.92- 3.57) | 47 |  | **3.99 (1.96- 8.11)** |  |

Possible heart failure (HF) defined as:

1. Baseline BNP> 125 if a participant is younger than 75, or BNP > 450 if ≥ 75 years or older

a. N=362 among first subcohort (random sample, plus participants with CHD events)

b. N=297 among second subcohort (random sample, plus participants with MI events)

2. Participants who had an incident MI event with concurrent prevalent HF (n=18) and participants with MI and incident HF at the same date (n = 32)

3. Participants who had a first HF admission before an incident MI (n=2)

Participants without MI event, but with incident HF before 01/01/2011 were censored at the time of first HF admission

Model 1 = BNP alone

Model 2 = Model 1 and Demographics (age, race, sex, household income, education, geographic region of residence), additional Framingham risk factors (current smoking status, systolic blood pressure, diabetes, HDL and total cholesterol) and other CVD risk factors and covariates (body mass index, log-transformed hsCRP, log-transformed ACR and medication use.

**Bold p <.05**

a. Cause specific model using Lunn and McNeil approach for competing risk analyses.

.
